# Selective Cytopheretic Device Use in Continuous Kidney Replacement Therapy in Children: A Cohort Study With a Historical Comparator

**DOI:** 10.1016/j.xkme.2024.100792

**Published:** 2024-02-15

**Authors:** Stuart L. Goldstein, Nicholas J. Ollberding, David J. Askenazi, Rajit K. Basu, David T. Selewski, Kelli A. Krallman, Lenar Yessayan, Harvey David Humes

**Affiliations:** 1 Division of Nephrology & Hypertension, Cincinnati Children’s Hospital Medical Center, Cincinnati, Ohio; 2 Division of Biostatistics and Epidemiology, Cincinnati Children's Hospital Medical Center, Cincinnati, Ohio; 3 Division of Nephrology, University of Alabama at Birmingham, Birmingham, Alabama; 4 Division of Critical Care Medicine, Lurie Children’s Hospital of Chicago, Chicago, Illinois; 5 Division of Pediatric Nephrology, Medical University of South Carolina, Charleston, South Carolina; 6 Division of Nephrology, University of Michigan, Ann Arbor, Michigan

**Keywords:** Acute kidney injury, children, continuous renal replacement therapy, safety, selective cytopheretic device, survival

## Abstract

**Rationale and Objective:**

Critically ill children with acute kidney injury (AKI) requiring continuous renal replacement therapy (CRRT) are at increased risk of death. The selective cytopheretic device (SCD) promotes an immunomodulatory effect at circuit-ionized calcium of <0.40 mmol/L. In an adult CRRT patient study, SCD-treated patients reported improved survival or dialysis independence. We reported safety data from children who received CRRT-SCD therapy and compared outcomes with a historic pediatric CRRT cohort.

**Study Design:**

We performed 2 prospective multicenter studies to evaluate the safety and feasibility of SCD in critically ill children.

**Setting and Participants:**

Four pediatric institutions enrolled children weighing 10 kg or more with AKI and multi-organ dysfunction receiving CRRT as the standard of care with the SCD-integrated post-CRRT membrane.

**Exposure:**

Patients received CRRT-SCD with regional citrate anticoagulation for up to 7-10 days, or CRRT discontinuation, whichever came first.

**Analytical Approach:**

We reported serious adverse events among patients and CRRT-SCD-related process and outcome variables. We compared survival to intensive care unit (ICU) discharge rates between the CRRT-SCD cohort and a matched cohort from the prospective pediatric CRRT registry, using odds ratios in multivariable analysis for factors associated with prospective pediatric CRRT patient ICU mortality. To validate these crude analyses, Bayesian logistic regression was performed to assess for attributable benefit-risk assessment of the SCD.

**Results:**

Twenty-two patients received CRRT-SCD treatments. Fifteen serious adverse events were recorded; none were SCD-related. Seventeen patients survived till ICU discharge or day 60. Both multivariable and Bayesian analyses revealed a probable benefit of the addition of SCD. Fourteen of the 16 patients surviving ICU discharge reported a normal estimated glomerular filtration rate and no patient was dialysis dependent at 60 days.

**Limitations:**

The study had a few limitations, such as (1) a small sample size in the SCD-PED cohort group; (2) unchanging historic control group; and (3) adverse events were not recorded in the control group.

**Conclusions:**

The SCD therapy is feasible, safe, and demonstrates probable benefit for critically ill children who require CRRT for AKI.

## Plain Language Summary

Only 50% of critically ill children with kidney failure who require the most advanced forms of dialysis survive. One cause of this poor survival is the increased activation of the immune system, which leads to inflammation and organ failure. Reducing the effects of inflammation could improve the survival rate in this very sick population. We studied a device, the selective cytopheretic device (SCD) that lessens the activity of cells in the body that cause inflammation. Twenty-two children received treatment with the SCD put in line with a standard dialysis machine, of which 17 (77%) survived (compared to the expected 11). There were no adverse effects noted with the SCD. Hence, we suggest that the SCD offers an option to improve outcomes in critically ill children with kidney failure.

Acute kidney injury (AKI) is a significant complication in critically ill children, as it results in increased morbidity and mortality.^1^, ^2^, ^3^, ^4^ Acute kidney injury develops predominantly because of the injury or necrosis of renal proximal tubule cells, often concurrently with sepsis. Part of the disease process in patients with AKI is often the development of a systemic inflammatory response syndrome, resulting in cardiovascular collapse, ischemic damage to vital organs, and multi-organ dysfunction.^5^^,^^6^

Despite advancements in renal replacement therapy (RRT) and intensive care medicine practice over the past 2 decades, mortality rates in children with AKI and multi-organ dysfunction requiring continuous RRT (CRRT) have not improved, remaining at 50%.^7^, ^8^, ^9^ Children who survive an AKI episode are at increased risk of chronic kidney disease (CKD).^10^, ^11^, ^12^ Because activated leukocytes are central to the pathogenesis or progression of septic shock and other clinical inflammatory disorders, new therapeutic approaches are being considered to limit the deleterious clinical effects of activated leukocytes.^13^

The selective cytopheretic device (SCD, SeaStar Medical, Inc), immunomodulates activated circulating leukocytes and provides a new therapeutic approach to systemic inflammatory response syndrome and AKI in the setting of a low ionized calcium (iCa^2+^) environment. The SCD’s low shear blood flow path allows activated circulating neutrophils and monocytes to bind to the SCD’s biocompatible membrane. Higher activation of leukocytes (higher density of binding molecules, integrins or CD11b, present on the leukocyte) results in a higher degree of binding on the SCD.^14^ For monocytes, the SCD’s controlled microenvironment aids in the binding of the most proinflammatory circulating subsets while allowing less proinflammatory phenotypes to remain in the patient’s systemic circulation. This SCD-promoted selective sequestration results in a prolonged shift to a greater number of less inflammatory (reparative or patrolling) circulating monocytes in the evolving inflammatory state during SCD therapy.^15^ Because monocytes migrate from the circulation into tissues, this functional phenotype shifts to circulating monocytes would promote an earlier transition from M1 (degradative) to M2 (reparative) macrophages in damaged tissue. For neutrophils, the SCD-controlled microenvironment promotes the apoptotic program in the bound neutrophils and leads to cell senescence. This change results in the release of the apoptotic neutrophils back to the systemic circulation.^14^

We have previously published SCD results from the following: (1) a prospective US Food and Drug Administration (FDA) funded, multicenter safety and feasibility study^16^ in children weighing 20 kg or more; (2) a case-report^17^ of a 21-month-old with Epstein-Barr virus driven hemophagocytic lymphohistiocytosis and; (3) a case series of 3 children with Shiga-toxin producing *Escherichia coli* associated hemolytic uremic syndrome (STEC-HUS).^18^ We now compile data from all these patients and others into a single report to summarize the entire pediatric patient experience with the SCD and compare patient-centered outcomes with the unchanging historic CRRT patient intensive care unit (ICU) survival rate of 50%. Our aim is to support the premise that SCD treatment is safe, with probable benefit in this critically ill pediatric population, which is required by the FDA to grant a Humanitarian Device Exemption (HDE) for marketing a device in the United States for treatment of a condition that affects not more than 8,000 patients per year.

## Methods

We performed 2 prospective studies (SCD-PED-01 and SCD-PED-02) at 4 centers (Cincinnati Children’s Hospital Medical Center, University of Michigan/ CS Mott Children’s Hospital, the University of Alabama at Birmingham/ Children’s of Alabama, and Emory University/Children’s Health Care of Atlanta at Egleston) from 2016 to 2022. Children weighing 20 kg or more and up to 22 years of age (SCD-PED-01), and ≥10-20 kg (SCD-PED-02) admitted to an ICU were screened for eligibility. Individuals who had AKI, as defined by the Kidney Disease Improving Global Outcomes (KDIGO) criteria^19^ and multi-organ dysfunction syndrome receiving CRRT as part of the standard of clinical care, were eligible to be enrolled. All patients receiving CRRT at a study site were screened locally and consecutively for inclusion and exclusion criteria. Parents of patients who passed local screening were approached for written consent only after one of the overall study principal investigators (SCE-PED-01: HDH and SLG; SCD-PED-02: SLG and LY) agreed with the site enrollment eligibility assessment. No other criteria were used to enroll versus not enroll in the study. Multi-organ dysfunction syndrome was defined as a respiratory disease requiring invasive mechanical ventilation or cardiovascular compromise requiring the provision of a continuous infusion of an inotropic or vasoactive medication. Pediatric Risk of Mortality score (PRISM III) at ICU admission was used to assess the severity of patient’s illness.^20^ The institutional review board at each center approved the study before patient enrollment at their site. Written informed consent was obtained from the participant’s parents or person with medical decision-making power before participant enrollment.

The studies received an IDE (G120174) from the US FDA and were registered at www.clinicaltrials.gov (SCD-PED-01: NCT02820350, SCD-PED-02: NCT04869787) before each study’s commencement. The SCD-PED-01 was funded mostly by an Office of Orphan Products Development (OOPD) grant (R01FD005092) from the US FDA with a small subsequent grant from SeaStar Medical, Inc to complete the final 2 participants’ enrollment. The SCD-PED-02 was funded by the Frankel Innovation Initiative at the University of Michigan. Because these are primarily safety studies (adverse events (AE) and serious adverse events (SAE), results and progress were reviewed by an external data safety monitoring board at least annually and within 5 days for any SAE.

### CRRT-SCD protocol

**Figure 1:**
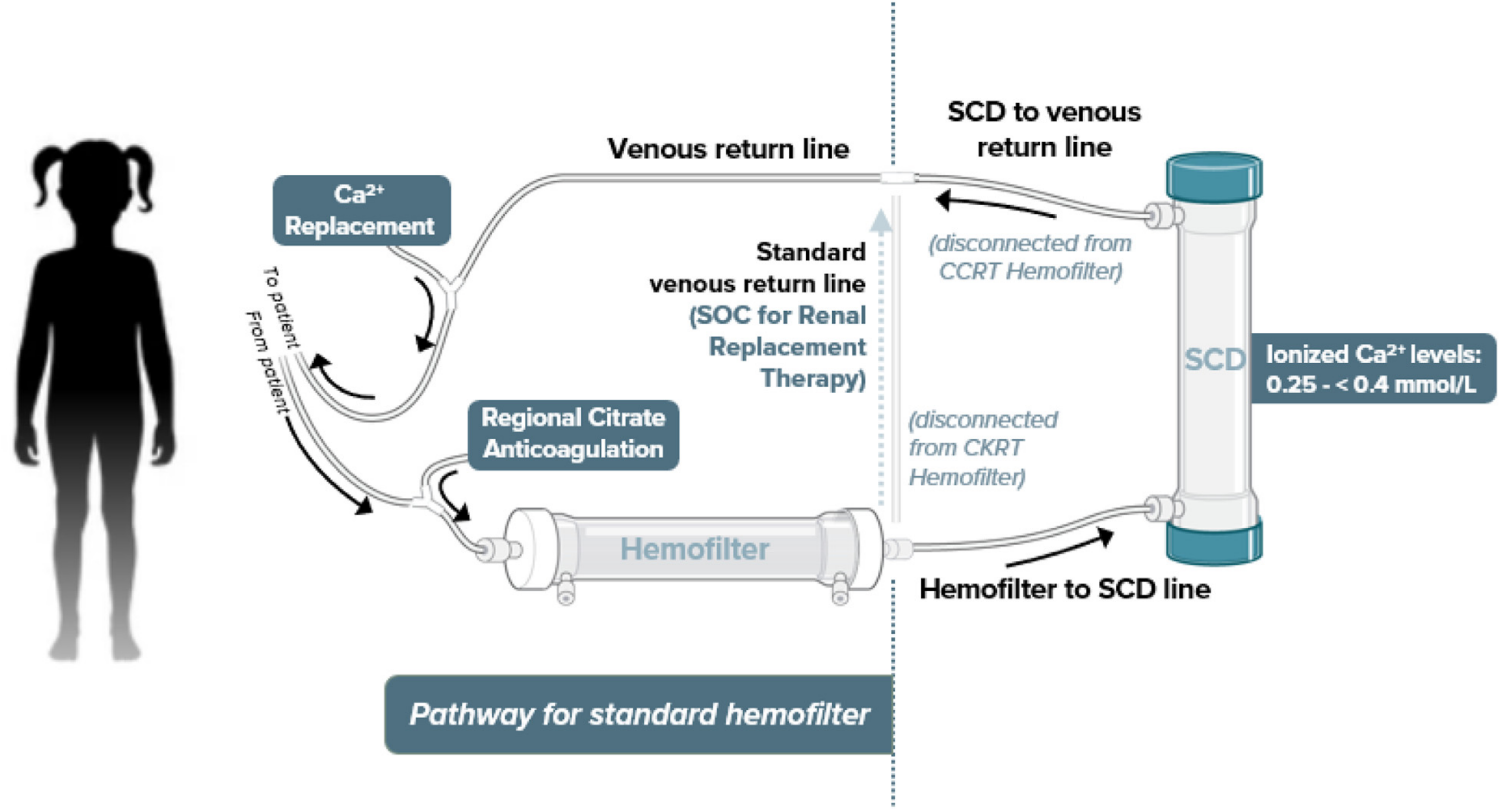
CRRT-SCD circuit configuration showing the blood flow path from the patient to the CRRT filter with the SCD-integrated post-CRRT filter. CRRT, continuous renal replacement therapy; SCD, selective cytopheretic device.

**Figure 2:**
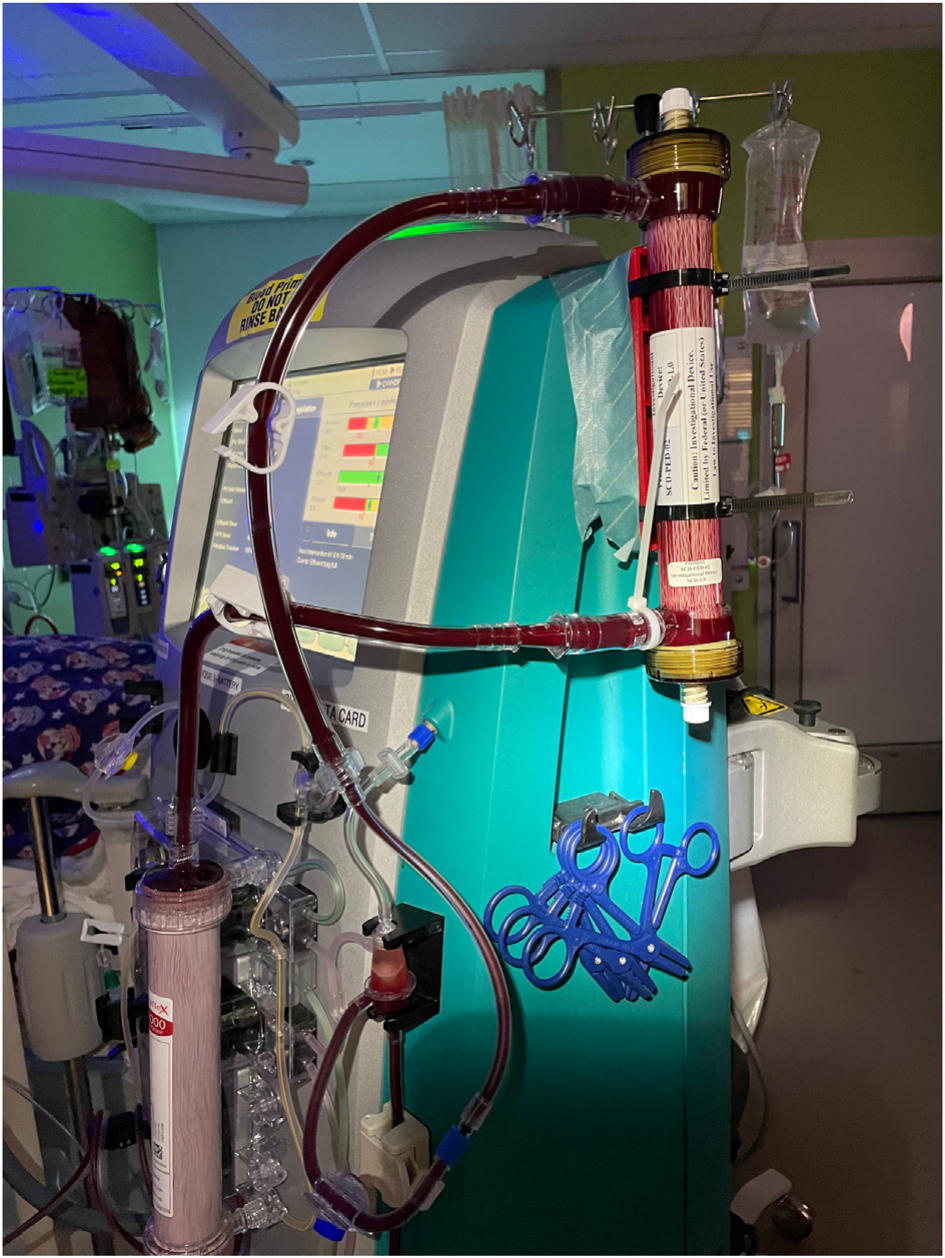
Photograph of the SCD-integrated post-CRRT during a CRRT-SCD procedure. Note: The device shown may not represent final future device configuration. CRRT, continuous renal replacement therapy; SCD, selective cytopheretic device.

The pediatric SCD protocol has been published extensively elsewhere.^16^ All centers provided CRRT with regional citrate anticoagulation as part of their local standard of clinical care with the following study required constraints: (1) small solute clearance was required to be at least 2,000 mL/hour/1.73 m^2^ of patient body surface area prescribed as total CRRT effluent rate; (2) regional citrate anticoagulation was provided with a well-described protocol^21^, ^22^, ^23^ using anticoagulant dextrose-A to maintain CRRT circuit iCa^2+^ of <0.40 mmol/L for at least 90% of the time a patient was receiving CRRT-SCD and a continuous intravenous calcium infusion to maintain patient systemic iCa^2+^ of >1.0 mmol/L; (3) CRRT circuit and patient systemic iCa^2+^ were measured at least every 6 hours; and (4) only polysulfone based CRRT membranes could be used (ie, no polyacrylonitrile membranes). The SCD filter is integrated into the CRRT circuit post-CRRT membrane (Figs 1 and 2). The SCD was changed daily for up to 7 days (SCD-PED-01), 10 days (SCD-PED-02), or CRRT discontinuation, whichever occurred first.

### Analyses

The primary outcome of these studies was to evaluate the safety of the SCD in children weighing 10 kg or more to support an application for an HDE. Participant demographic data and CRRT/SCD data are aggregated and reported as median and interquartile range (IQR). The AEs and SAEs were reported by the site investigators based on predetermined criteria. The site investigator determined if any AE was related to the device. An AE, whether considered study-treatment related or not, which fit any of the criteria below, was considered an SAE:

• Resulted in death
• Was life-threatening (meaning that the patient was at risk of death at the time of the event; this does not refer to an event that might have caused death if it had occurred in a more severe form)
• Required in-patient hospitalization or prolongs the existing hospitalization
• Was a persistent disability or incapacity
• Was considered an important medical event by the investigator (eg, surgery, return to ICU, or emergency procedures)

The primary probable efficacy outcome was patient survival to ICU discharge or to 60 days after CRRT (CRRT-SCD) initiation (whichever came first). Secondary outcomes were estimated glomerular filtration rate (eGFR) at 28 and 60 days after CRRT-SCD initiation. The serum creatinine-based bedside Schwartz equation^24^ was used to calculate the eGFR:

$$\text{eGFR}\;(\text{mL}/\min/1.73\;m^{2}=0.413\times \text{patient}\;\text{height}\;\left[\text{cm}\right]/\text{serum}\;\text{creatinine}\;\left[\text{mg}/\text{dL}\right])\text{.}$$

To assess for probable benefit, patient survival to ICU discharge or 60 days in the combined cohort was compared with a subset from the prospective pediatric continuous renal replacement therapy (ppCRRT) Registry.^7^ The ppCRRT registry enrolled patients from 2001 to 2005 at 13 centers in the United States. A total of 370 patients were enrolled in the ppCRRT registry. A subset of the ppCRRT cohort that was used for comparison were patients who weighed > 10 kg at ICU admission and were receiving invasive mechanical ventilation or 1 vasoactive medication at the time of CRRT initiation. The final comparison subset comprised 210 patients who met the matching criteria and had complete data.

### Statistical Analysis

All demographic, CRRT-related and outcome data are presented as median (IQR) or rates where appropriate. The ppCRRT did not collect data after ICU discharge, so a long-term comparative assessment could not be performed. In addition, the ppCRRT did not collect CRRT-related AEs or SAEs systematically, so adverse event rates could not be compared between the 2 cohorts.

Potential differences in demographic, CRRT treatment and patient outcome data between the SCD-PED and ppCRRT cohorts were compared with the Kruskal-Wallis test, or by χ^2^ analysis with the Fisher exact test for categorical variables. Multivariable logistic regression analyses adjusted for published factors associated with ppCRRT patient ICU mortality including patient age, pediatric risk of mortality score II^25^ (PRISM II was used in the ppCRRT), and fluid accumulation from ICU admission to CRRT initiation were used to calculate adjusted odds ratios (aOR) for ICU survival.^7^ A sensitivity analysis excluded patients in the SCD-PED cohort who received extracorporeal membrane oxygenation (ECMO) because ECMO was an exclusion in the ppCRRT Registry. These analyses were performed with the Stata (Version 17, StatCorp, Inc).

To validate these crude analyses, Bayesian logistic regression, as implemented by the brms package (version 2.17.0), was used to estimate the probability that the log odds of survival to 60 days in the SCD cohort exceeded that observed in the ppCRRT cohort. Models were fit assuming default flat (eg, uniform) priors and a logit link function to model the Bernoulli distributed responses. A total of 8,000 iterations were performed across 4 chains with a burn-in of 500 iterations per chain using the STAN No-U-Turn Sampler and model convergence was assessed by trace plots and r-hat values. Posterior samples were transformed to the probability scale using the inverse logit transform and used to compute the predicted probability of surviving to 60 days for each cohort, the probability of treatment benefit (eg, log odds SCD > ppCRRT), and the predicted risk difference. All analyses were performed using the R software environment for statistical computing and graphics (version 4.1.1) and rstan (version 2.21.3).

## Results

**Figure 3:**
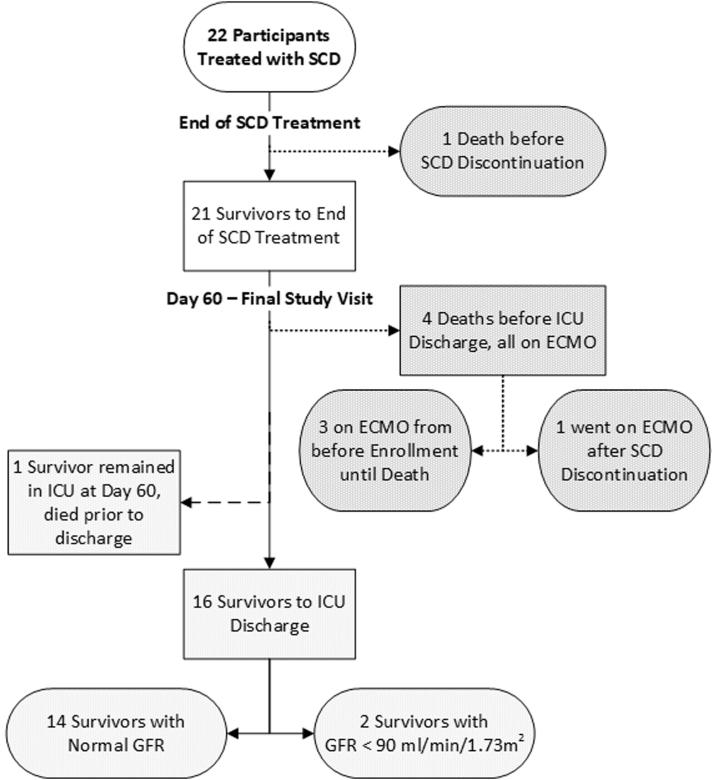
Flow and outcomes for participants enrolled in the SCD-PED-01 and SCD-PED-02 studies. SCD, selective cytopheretic device.

The pre-SCD initiation, SCD procedure and patient outcome data are shown in aggregate for the 22 patients and divided between the 2 studies in Table 1. The aggregate patient outcomes from CRRT-SCD initiation to day 60 postinitiation are depicted in Fig 3.

**Table 1:** Patient Demographics and Pre-SCD Characteristics

Characteristics	Combined (n = 22)	SCD-PED-01 (n = 16)	SCD-PED-02 (n = 6)
**Patient sex (F/M) (%)**	10 (45.4%)/12 (54.5%)	8 (50%)/8 (50%)	2 (33.3%)/4 (66.7%)
**Patient age (y)**	9.5 (4.3-15.8)	12.1 (7.8-16.8)	2.1 (1.7-3.5)
**Patient weight (kg)**	30.3 (16.5-62.1)	53.3 (27.1-69.5)	13.4 (12-14.1)
**PRISM III at ICU admission**	9.5 (7-14)	10.5 (6.5-14.5)	8.5 (7-13)
**Mechanical ventilation at SCD Start**	21 (95.4%)	15 (93.8%)	6 (100%)
**Vasoactive medication at SCD start**	14 (63.6%)	9 (56.2%)	5 (83.3%)
**ECMO at SCD Initiation**	3 (13.6%)	2 (12.5%)	1 (16.7%)
**ECMO at SCD initiation or After**	4 (18.2%)	3 (18.8%)	1 (16.7%)
**Sepsis at SCD initiation**	15 (68.2%)	10 (62.5%)	5 (83.3%)
**Fluid accumulation to CRRT Start (%)**	7.5 (3.0-14.5)	5.1 (2.8-9.7)	23.6 (12.5-31.6)
**ICU fays to CRRT Start**	3.5 (1-5)	2 (1-4.5)	4.5 (4-8)

CRRT-SCD procedure characteristics			
	Combined (n = 22)	SCD-PED-01 (n = 16)	SCD-PED-02 (n = 6)
**CRRT days before SCD**	4 (0-4.8)	4 (0-4.5)	7.8 (0-25.8)
**Days on CRRT-SCD**	6 (3-7)	6 (4-7)^a^	5 (1-9)^b^
**CRRT days after SCD**	0 (0-4)	0 (0-4.5)	0 (0-0)
**SAEs total**	15	9	6

CRRT-SCD patient outcomes			
	Combined	SCD-PED-01	SCD-PED-02
**Survival to SCD discontinuation**	21 (95.4%)	15 (93.8%)	6 (100%)
**Survival to CRRT discontinuation**	21 (95.4%)	12 (75%)	5 (83.3%)
**Survival to ICU discharge**^c^	16 (72.7%)	12 (75%)	4 (75%)
**Survival to 60 days**^c^	17 (77.3%)	12 (75%)	5 (83.3%)
**Survival to ICU discharge or 60 days in non-ECMO patients**	17 (94.4%)	11 (91.4%)	6 (100%)
**Day 28 estimated GFR (mL/min/1.73** **m**^**2**^**)**	90 (45-140)	80 (37-109)	104 (80-240)
**Day 60 estimated GFR (mL/min/1.73** **m**^**2**^**)**	105 (97-119)	105 (99-114)	111 (95-180)

*Notes:* All values are n (%) or median (interquartile range); SCD-PED-01 protocol had maximum SCD treatment of 7 days.

Abbreviations: ECMO, extracorporeal membrane oxygenation; PRISM, pediatric risk of mortality score; SCD, selective cytopheretic device.

a SCD-PED-02 protocol had maximum SCD treatment of 10 days.

b One patient alive and still in ICU at 60 days.

c All 4 patients receiving ECMO during their ICU course died.

All but 1 patient survived until the time SCD and CRRT discontinuation. The 1 deceased patient (reported previously)^16^ in SCD-PED-01 died 7 hours after CRRT-SCD initiation and was found to have extensive invasive viral myocarditis on biopsy. The data safety monitoring board and FDA each reviewed the case and assessed the death to not be related to the SCD. Seventeen patients survived to ICU discharge or day 60; 16 patients survived until the time of ICU discharge, and 1 was alive in the ICU at day 60 but died before ICU discharge. Four of the 5 nonsurvivors received ECMO support at some point during their ICU course (3 while on CRRT-SCD and 1 after CRRT-SCD completion). Fourteen of 16 ICU survivors had an eGFR of >90 mL/min/1.73 m^2^ and no patient was dialysis dependent on day 60 after the CRRT-SCD initiation. Adverse and serious adverse events are listed in Tables 2 and 3. A total of 20 SAEs were reported in 10 patients across the 2 studies, none were considered device-related. A total of 69 AEs were reported across the 2 studies, 18 of which were classified as severe, none of which were considered to be device-related.

**Table 2:** Serious Adverse Events in SCD-PED-01

Patient #	Adverse Event	Severity	Device Related
1	Cardiorespiratory arrest	Severe	No
2	Pneumoperitoneum	Severe	No
2	Nephrolithiasis	Moderate	No
3	Stevens-Johnson syndrome	Severe	No
4	Cardiac arrest	Severe	No
5	Junctional tachycardia	Severe	No
5	Vascular graft occlusion	Moderate	No
5	Worsening respiratory failure	Severe	No
6	Cerebral hemorrhage	Severe	No
7	Cardiac arrest	Severe	No
8	Pulmonary hemorrhage	Severe	No
8	Adrenal insufficiency	Severe	No

Serious Adverse Events in Study SCD-PED-02			
Patient #	Adverse event	Severity	Device related
1	Pneumothorax	Severe	No
1	Medical device site thrombosis	Severe	No
1	Cardiac arrest	Severe	No
1	Septic shock	Severe	No
2	Acute respiratory failure	Severe	No
2	Lactic acidosis	Moderate	No
**Total**		**18 SAEs in 10 patients: none were device-related**	

Abbreviations: SCD, selective cytopheretic device.

**Table 3:** Adverse Events in SCD-PED-01

System Organ Class	Severity			Total
Adverse Event Term	Mild	Moderate	Severe	
**Blood and lymphatic system disorders**	1	2		3
Thrombocytopenia		1		1
Thrombocytosis	1			1
Heparin-induced thrombocytopenia		1		1
**Cardiac disorders**	3		3	6
Cardiac arrest			1	1
Junctional tachycardia			1	1
Tachycardia	3			3
Ventricular arrhythmias and cardiac arrest			1	1
**Gastrointestinal disorders**			**1**	**1**
Pneumoperitoneum			1	1
**General disorders and administration site conditions**	5			5
Hypothermia	3			
Pyrexia	2			
**Infections and infestations**		1	1	2
Postprocedure pneumonia (hospital-acquired)		1		1
Stevens-Johnson syndrome			1	1
**Injury, poisoning, and procedural complications**		1		1
Subcutaneous emphysema		1		1
**Metabolism and nutritional disorders**	1	4	1	6
Adrenal insufficiency			1	1
Hyperglycemia		3		3
Hypokalemia	1	1		2
**Nervous system disorders**			1	1
Cerebral hemorrhage			1	1
**Psychiatric disorders**		1		1
Intensive care unit delirium		1		1
**Renal and urinary disorders**		1		1
Nephrolithiasis		1		1
**Respiratory, thoracic, and mediastinal disorders**		2	2	4
Acute respiratory failure		2	1	3
Cardiorespiratory arrest			1	1
**Surgical and medical procedures**		1		1
Vascular graft occlusion		1		1
**Vascular disorders**	6		2	8
Hypertension	1			1
Hypotension	5		1	6
Pulmonary hemorrhage			1	1
**Total**	16	13	11	40

System organ class	Severity			Total
Adverse event term	Mild	Moderate	Severe	
**Blood and lymphatic system disorders**	1	3	1	5
Anemia	1			1
Coagulopathy		1		1
Thrombocytopenia			1	3
**Cardiac disorders**			1	1
Cardiac arrest			1	1
**Hepatobiliary disorders**		2		2
Hyperbilirubinemia		2		2
**Infections and infestations**			1	1
Septic shock			1	1
**Injury, poisoning, and procedural complications**			1	1
Medical device site thrombosis			1	1
**Investigations**	3			3
Blood bicarbonate decreased	1			1
Blood fibrinogen decreased	1			1
White blood cell count increased	1			1
**Metabolism and nutrition disorders**	8	3	1	12
Hypercalcemia	1			1
Other electrolyte abnormality	6	1	1	8
Hypertriglyceridemia		1		1
Hypoalbuminemia	1			1
Lactic acidosis		1		1
**Nervous system disorders**	1			1
Cerebral dysfunction	1			1
**Product issues**	1			1
Device leakage	1			1
**Respiratory, thoracic, and mediastinal disorders**			2	2
Acute respiratory failure			1	1
Pneumothorax			1	1

Abbreviations: SCD, selective cytopheretic device.

Although no claims can be made regarding efficacy with such a small patient sample size, an HDE study must show a device to be safe with probable benefit for marketing clearance. To assess for probable benefit, we compared the combined cohort to a size matched cohort from the (ppCRRT) registry (Table 4). No differences were observed between the 2 cohorts with respect to patient characteristics or severity of illness at the time of CRRT initiation. One hundred fifteen of 210 patients from the matched ppCRRT cohort and 17 of the 22 patient SCD combined cohort survived to the time of ICU discharge or 60 days (55%; 95% CI, 48%-62% vs. 77%; 95% CI, 55%-92%; *P* = 0.04). Multivariable logistic regression analysis showed nonsignificant increased survival to ICU discharge or day 60 in the SCD-PED cohort (aOR 2.65 (0.93-7.53); *P* = 0.07), but increased survival when patients receiving ECMO in the SCD-PED cohort were removed from analysis (aOR, 13.4 (1.7-103); *P* = 0.01) (Tables 5 and 6).

**Table 4:** SCD and ppCRRT Population Comparisons

	ppCRRT (n = 210)	CRRT-SCD (n = 22)	*P*
**Patient sex (F)**	94 (44.7%)	10 (45.4%)	0.91
**Patient age (y)**	11.3 (5.0-16.4)	9.5 (4.3-15.8)	0.50
**Patient weight (kg)**	36.9 (19-60)	30.3 (16.5-62.1)	0.86
**PRISM II at ICU admission**	12 (8-18)	11.5 (5-15)	0.11
**Mechanical ventilation at CRRT start**	183 (87.1%)	21 (95.4%)	0.21
**Pressors at CRRT start**	160 (76.1%)	15 (68.1%)	0.48
**ICU fluid accumulation at CRRT start (%)**	8.0 (1.3-22.8)	7.5 (3.0-14.5)	0.42
**CRRT duration (d)**	7 (3-13)	6.5 (5-11.5)	0.88
**Survival to ICU discharge or day 60**	115 (54.8%)	17 (77.3%)	0.04

*Notes:* All comparisons are n (%) or median (interquartile range).

Abbreviations: ICU, intensive care unit; ppCRRT, prospective pediatric continuous renal replacement therapy; PRISM, pediatric risk of mortality score; SCD, selective cytopheretic device.

**Table 5:** Multivariable Logistic Regression Analysis of Predictors of Survival to ICU Discharge (all patients)

Analysis	Adjusted Odds Ratio (95% CI)	*P*
**Cohort (SCD-PED)**	2.65 (0.93-7.53)	0.07
**Patient age (y)**	0.99 (0.97-1.01)	0.32
**PRISM II at ICU admission**	1.00 (0.97-1.04)	0.99
**ICU fluid accumulation at CRRT start (%)**	0.99 (0.97-1.00)	0.13

Abbreviations: CRRT, continuous renal replacement therapy; ICU, intensive care unit; PRISM, pediatric risk of mortality score; SCD, selective cytopheretic device.

**Table 6:** Multivariable Logistic Regression Analysis of Predictors of Survival to ICU Discharge (excluding patients receiving extracorporeal membrane oxygenation)

Analysis	Adjusted Odds Ratio (95% CI)	*P*
**Cohort (SCD-PED)**	13.4 (1.7-103)	0.01
**Patient age (y)**	0.99 (0.97-1.01)	0.32
**PRISM II at ICU admission**	1.00 (0.97-1.04)	0.94
**ICU fluid accumulation at CRRT start (%)**	0.99 (0.97-1.00)	0.14

Abbreviations: CRRT, continuous renal replacement therapy; ICU, intensive care unit; PRISM, pediatric risk of mortality score; SCD, selective cytopheretic device.

**Figure 4:**
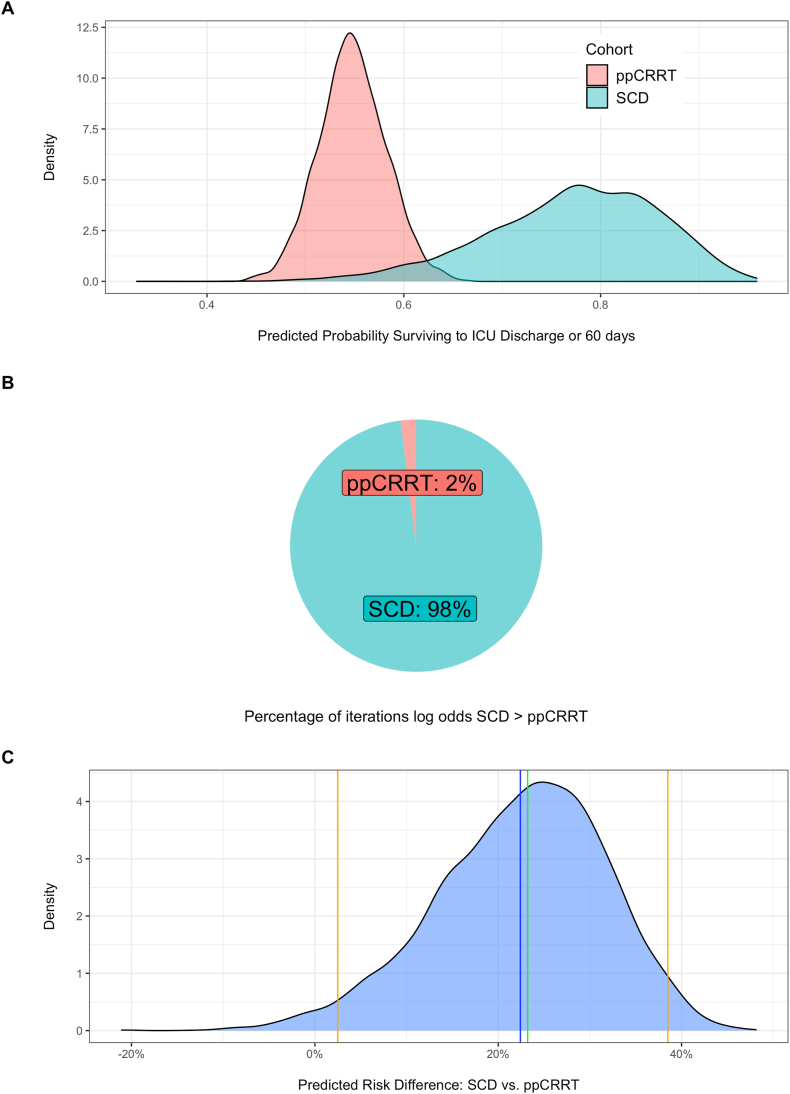
Predicted probabilities of survival to ICU discharge or 60 days. (A) Posterior predicted probabilities of survival to ICU discharge or 60 days for the SCD (blue) and ppCRRT (red) cohorts. (B) The percentage of posterior samples in which the log odds of surviving to ICU discharge or 60 days were greater for the SCD cohort than the ppCRRT Registry cohort. (C). Predicted risk difference computed from the posterior samples. The mean difference is shown in blue. The median difference is shown in green and 95% credible interval is shown in orange. ICU, intensive care unit; ppCRRT, prospective pediatric continuous renal replacement therapy; SCD, selective cytopheretic device.

To validate these comparisons, we undertook a Bayesian analysis comparing the 2 cohorts. The distribution of the predicted probabilities for survival to the time of ICU discharge or 60 days obtained from the posterior samples is shown in Fig 4A for each cohort separately. In 98% of the posterior samples, the log odds of surviving to the time of ICU discharge or 60 days were greater for the SCD when compared with the ppCRRT cohort (Fig 4B); highlighting the probable benefit in this cohort of patients. The predicted risk difference was 22.4% (95% credible interval, 2.5%-38.5%) for the SCD versus ppCRRT cohort (Fig 4C). A similar estimate of the probable benefit (99%) was obtained by directly estimating the percentage of times the mean of a binomially distributed sample of n = 22 observations with a probability of 17 of the 22 exceeded that of 1 drawn from a sample of n = 210 observations and probability of 115 of the 210 when taken over 1 × 10^6^ samples.

## Discussion

We report aggregate data from 2 pediatric studies using the SCD to support critically ill children with AKI, multi-organ dysfunction syndrome who received CRRT as part of their standard of care. Our data suggest that SCD therapy was safe and feasible in this cohort, as no device-related SAEs were observed, and that there was a high likelihood of benefit of SCD therapy in this critically ill pediatric population. Our data seem to support a previous adult pivotal trial, which demonstrated a decreased composite endpoint of death or dialysis dependence at day 60.^26^

The precise mechanism of action of the SCD is becoming better understood and appears to be an immunomodulatory process that inhibits leukocyte activation, a critical component of the systemic inflammatory response syndrome leading to multi-organ dysfunction syndrome. The modulation of the dysregulated inflammatory state also allows recovery of the kidney function in AKI and other associated organ failures. The cartridge acts as an SCD in the presence of citrate anticoagulant to bind and immunomodulate potentially damaging circulating leukocytes. This perspective is based on evolving data from in vitro bench studies, preclinical animal models, and human clinical trials using measurements of inflammatory biomarkers and leukocyte cell sorting and cytometric analysis. More complete discussions of the possible mechanisms can be found elsewhere.^14^^,^^27^^,^^28^

Given the limited sample size of 22 participants, we can make no claims about the efficacy of the SCD on patient-related outcomes. However, the 77% ICU survival rate observed compares favorably to published CRRT studies in critically ill children with multi-organ dysfunction syndrome that show a survival rate of about 50%.^7^, ^8^, ^9^^,^^29^^,^^30^ The largest multicenter study from the ppCRRT registry who received mechanical ventilation or a vasoactive agent, which were identical inclusion of SCD-PED-01 and SCD-PED-02 showed an ICU survival rate of 51.7%.^9^ Notably, ECMO provision was an exclusion in the ppCRRT, so a potentially more relevant comparison is a combined SCD ICU survival rate of 16 of the 17 (94.1% excluding the 4 patients receiving ECMO in the CRRT-SCD cohort) vs. 51.7% in the ppCRRT cohort. In addition, our analyses comparing survival to the time of ICU discharge or 60 days for patients using the SCD versus those receiving mechanical ventilation or a vasoactive agent in the ppCRRT registry suggests a potential probability of treatment benefit as high as 98% for those receiving SCD. These results are sufficient to support a probable benefit for this device when compared with the standard treatment, which is a required component for HDE clearance by the US FDA. In part, based on the clinical data contained herein, the FDA approved the HDE for the SCD with the indication “to treat acute kidney injury (AKI) in pediatric patients (weight ≥10 kg and age ≤22 years) with acute kidney injury (AKI) due to sepsis or a septic condition on antibiotic therapy and requiring renal replacement therapy (RRT)”.^31^

Clearly, more information will be required to further substantiate the results presented herein. As part of the HDE commitment, a post-market registry study will be established to collect additional data regarding safety and patient-centered outcomes in the pediatric population. Because with recent advances in neonatal CRRT technology, work is underway to develop a miniaturized version of the SCD to address the needs of the population less than 10 kg in size. Finally, a randomized controlled pivotal study, neutrophil and monocyte deactivation by the SeLective CytopheretIc Device—a randomized clinical trial in acute kidney injury (NEUTRALIZE-AKI) plans to enroll 200 adult patients to assess whether the SCD will improve survival and reduce dialysis dependence after AKI.^32^

In summary, we demonstrated a high level of safety of SCD therapy and probable efficacy in children weighing 10 kg or more with AKI and multi-organ dysfunction receiving CRRT standard of care and suggest a favorable benefit-to-risk ratio in this critically ill pediatric population. The US FDA Office or orphan products development granted SeaStar Medical, Inc a humanitarian use designation for the SCD under the study indicated use. Further studies will be required to establish safety in smaller children, feasibility of integration with other CRRT platforms, and to demonstrate improved patient outcomes compared with current supportive therapy that is associated with a high rate of CKD in survivors.

## References

[1] Kaddourah A., Basu R.K., Bagshaw S.M., Goldstein S.L., AWARE Investigators (2017). Epidemiology of acute kidney injury in critically ill children and young adults. N Engl J Med.

[2] Jetton J.G., Boohaker L.J., Sethi S.K. (2017). Incidence and outcomes of neonatal acute kidney injury (AWAKEN): a multicentre, multinational, observational cohort study. Lancet Child Adolesc Health.

[3] Sutherland S.M., Ji J., Sheikhi F.H. (2013). AKI in hospitalized children: epidemiology and clinical associations in a national cohort. Clin J Am Soc Nephrol.

[4] Sutherland S.M., Byrnes J.J., Kothari M. (2015). AKI in hospitalized children: comparing the pRIFLE, AKIN, and KDIGO definitions. Clin J Am Soc Nephrol.

[5] Humes H.D., Fissell W.H., Weitzel W.F. (2002). The bioartificial kidney in the treatment of acute renal failure. Kidney Int Suppl.

[6] Humes H.D. (2000). Bioartificial kidney for full renal replacement therapy. Semin Nephrol.

[7] Symons J.M., Chua A.N., Somers M.J. (2007). Demographic characteristics of pediatric continuous renal replacement therapy: a report of the prospective pediatric continuous renal replacement therapy registry. Clin J Am Soc Nephrol.

[8] Modem V., Thompson M., Gollhofer D., Dhar A.V., Quigley R. (2014). Timing of continuous renal replacement therapy and mortality in critically ill children. Crit Care Med.

[9] Goldstein S.L., Somers M.J., Baum M.A. (2005). Pediatric patients with multi-organ dysfunction syndrome receiving continuous renal replacement therapy. Kidney Int.

[10] Menon S., Kirkendall E.S., Nguyen H., Goldstein S.L. (2014). Acute kidney injury associated with high nephrotoxic medication exposure leads to chronic kidney disease after 6 months. J Pediatr.

[11] Mammen C., Al Abbas A., Skippen P. (2012). Long-term risk of CKD in children surviving episodes of acute kidney injury in the intensive care unit: a prospective cohort study. Am J Kidney Dis.

[12] Madsen N.L., Goldstein S.L., Froslev T., Christiansen C.F., Olsen M. (2017). Cardiac surgery in patients with congenital heart disease is associated with acute kidney injury and the risk of chronic kidney disease. Kidney Int.

[13] Kaneider N.C., Leger A.J., Kuliopulos A. (2006). Therapeutic targeting of molecules involved in leukocyte-endothelial cell interactions. FEBS J.

[14] Ding F., Song J.H., Jung J.Y. (2011). A biomimetic membrane device that modulates the excessive inflammatory response to sepsis. PLOS ONE.

[15] Szamosfalvi B., Westover A., Buffington D., Yevzlin A., Humes H.D. (2016). Immunomodulatory device promotes a shift of circulating monocytes to a less inflammatory phenotype in chronic hemodialysis patients. ASAIO J.

[16] Goldstein S.L., Askenazi D.J., Basu R.K. (2021). Use of the selective cytopheretic device in critically ill children. Kidney Int Rep.

[17] Goldstein S.L., Yessayan L.T., Krallman K.A. (2023). Use of extracorporeal immunomodulation in a toddler with hemophagocytic lymphohistiocytosis and multisystem organ failure. Pediatr Nephrol.

[18] Hambrick H.R., Short K., Askenazi D. (2023). Hemolytic uremic syndrome-induced acute kidney injury treated via immunomodulation with the selective cytopheretic device. Blood Purif.

[19] (2013). KDIGO 2012 Clinical Practice Guideline for the Evaluation and Management of Chronic Kidney Disease. Kidney Int Suppl.

[20] Pollack M.M., Patel K.M., Ruttimann U.E. (1996). PRISM III: an updated pediatric risk of mortality score. Crit Care Med.

[21] Bunchman T.E., Maxvold N.J., Barnett J., Hutchings A., Benfield M.R. (2002). Pediatric hemofiltration: normocarb dialysate solution with citrate anticoagulation. Pediatr Nephrol.

[22] Bunchman T.E., Maxvold N.J., Brophy P.D. (2003). Pediatric convective hemofiltration: normocarb replacement fluid and citrate anticoagulation. Am J Kidney Dis.

[23] Brophy P.D., Somers M.J., Baum M.A. (2005). Multi-centre evaluation of anticoagulation in patients receiving continuous renal replacement therapy (CRRT). Nephrol Dial Transplant.

[24] Schwartz G.J., Munoz A., Schneider M.F. (2009). New equations to estimate GFR in children with CKD. J Am Soc Nephrol.

[25] Pollack M.M., Ruttimann U.E., Getson P.R. (1988). Pediatric risk of mortality (PRISM) score. Crit Care Med.

[26] Tumlin J.A., Galphin C.M., Tolwani A.J. (2015). A Multi-center, randomized, controlled, pivotal study to assess the safety and efficacy of a selective cytopheretic device in patients with acute kidney injury. PLOS ONE.

[27] Pino C.J., Westover A.J., Johnston K.A., Buffington D.A., Humes H.D. (2018). Regenerative medicine and immunomodulatory therapy: insights from the kidney, heart, brain, and lung. Kidney Int Rep.

[28] Selewski D.T., Goldstein S.L., Fraser E. (2017). Immunomodulatory device therapy in a pediatric patient with acute kidney injury and Multiorgan Dysfunction.multiorgan dysfunction. Kidney Int Rep.

[29] Hayes L.W., Oster R.A., Tofil N.M., Tolwani A.J. (2009). Outcomes of critically ill children requiring continuous renal replacement therapy. J Crit Care.

[30] Menon S., Arikan A.A., Fuhrman D.Y. (2023). Clinical characteristics of patients receiving continuous kidney replacement therapy: preliminary report from the worldwide exploration of renal replacement outcomes collaborative in kidney disease (WE-ROCK) (PAKI 4 Abstract 041). Ped Nephrolgy.

[31] https://www.fda.gov/media/176511/download?attachment.

[32] Yessayan L., Humes H.D., Scribe E.C., Iyer S.P.N., Chung K.K. (2024). Rationale and design of NEUTRALIZE-AKI: a multicenter, randomized, controlled, pivotal study to assess the safety and efficacy of a selective cytopheretic device in patients with acute kidney injury requiring continuous kidney replacement therapy. Nephron.

